# Upregulation of FLG, LOR, and IVL Expression by *Rhodiola crenulata* Root Extract via Aryl Hydrocarbon Receptor: Differential Involvement of OVOL1

**DOI:** 10.3390/ijms19061654

**Published:** 2018-06-04

**Authors:** Akiko Hashimoto-Hachiya, Gaku Tsuji, Mika Murai, Xianghong Yan, Masutaka Furue

**Affiliations:** 1 Department of Dermatology, Graduate School of Medical Sciences, Kyushu University, Fukuoka 812-8582, Japan; ahachi@dermatol.med.kyushu-u.ac.jp (A.H.-H.); gakku@dermatol.med.kyushu-u.ac.jp (G.T.); mika-m@dermatol.med.kyushu-u.ac.jp (M.M.); 2 P&G Innovation Godo Kaisha, Kobe 658-0032, Japan; yan.xh@pg.com; 3 Research and Clinical Center for Yusho and Dioxin, Kyushu University, Fukuoka 812-8582, Japan; 4 Division of Skin Surface Sensing, Graduate School of Medical Sciences, Kyushu University, Fukuoka 812-8582, Japan

**Keywords:** *Rhodiola crenulata* root extract, aryl hydrocarbon receptor, filaggrin, loricrin, involucrin, OVOL1

## Abstract

*Rhodiola* species are antioxidative, salubrious plants that are known to inhibit oxidative stress induced by ultraviolet and γ-radiation in epidermal keratinocytes. As certain phytochemicals activate aryl hydrocarbon receptors (AHR) or OVO-like 1 (OVOL1) to upregulate the expression of epidermal barrier proteins such as filaggrin (FLG), loricrin (LOR), and involucrin (IVL), we investigated such regulation by *Rhodiola crenulata* root extract (RCE). We demonstrated that RCE induced FLG and LOR upregulation in an AHR-OVOL1-dependent fashion. However, RCE-mediated IVL upregulation was AHR-dependent but OVOL1-independent. Coordinated upregulation of skin barrier proteins by RCE via AHR may be beneficial in the management of barrier-disrupted inflammatory skin diseases such as atopic dermatitis.

## 1. Introduction

Skin, the outermost part of the body, protects inner living tissues by forming an epidermal barrier organized by multiple barrier proteins [1]. Aryl hydrocarbon receptor (AHR) is a xenobiotic chemical sensor and is activated by various external and internal ligands such as dioxins, phytochemicals, and food metabolites [2,3,4]. Epidermal keratinocytes abundantly express AHR [2,3]. Upon ligand binding, the activated AHR translocates from the cytoplasm into the nucleus. This translocated AHR binds to its specific DNA recognition site, namely a xenobiotic-responsive element, and upregulates the transcription of responsive genes such as cytochrome P450 1A1 (CYP1A1) and epidermal barrier proteins including filaggrin (FLG), loricrin (LOR), and involucrin (IVL) [5,6,7]. As the barrier function is significantly disrupted in AHR-null mice, AHR plays a pivotal role in skin barrier integrity [8].

Proliferating basal keratinocytes commit to epidermal differentiation by exiting the cell cycle and migrating towards the skin surface, finally leading to the formation of anucleated corneocytes [1]. Corneocytes are composed of polymerized keratin filaments and a thick cell membrane called a cornified envelope. Desmosomes are the sites of initiation of the cornified envelope, where IVL molecules first become attached to plakins. Thereafter, FLG, LOR, and other barrier proteins are crosslinked to IVL to form the mature cornified envelope [1]. Notably, the expression of FLG, LOR, and IVL is upregulated by various AHR agonists such as coal tar, soybean tar glyteer, and *Galactomyces* fermentation filtrate [7,9,10]. In addition, our recent study proved that AHR-induced FLG and LOR upregulation is mediated by the OVO-like 1 (OVOL1) transcription factor [11,12]. OVOL1 is a key regulatory molecule that inhibits the proliferation and induces terminal differentiation of keratinocytes [13,14,15,16].

*Rhodiola* species are antioxidative, salubrious plants that are known to inhibit oxidative stress induced by ultraviolet and γ-radiation in epidermal keratinocytes [17,18]. Like other phytochemical extracts [4], *Rhodiola crenulata* extract (RCE) contains several AHR agonists including luteolin quercitrin, and isoquercitrin [19]. In this study, we found that RCE upregulated FLG and LOR expression in an AHR-OVOL1-dependent manner. However, AHR-mediated IVL upregulation was independent of the OVOL1 signal.

## 2. Results

### 2.1. RCE Is an AHR Agonist

**Figure 1:**
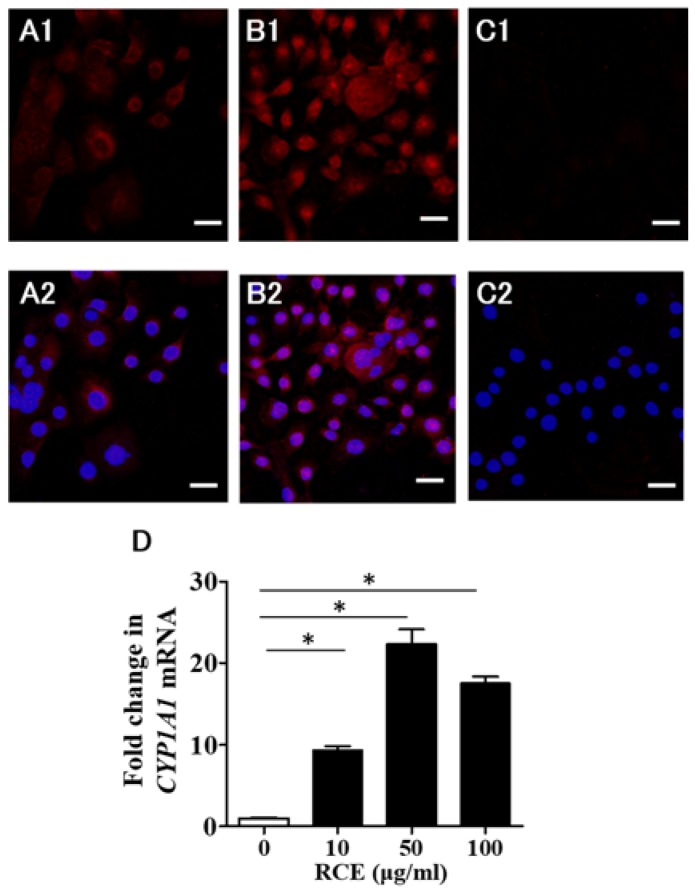
Immunolocalization of an aryl hydrocarbon receptor (AHR). (**A1**) AHR is mainly localized in the cytoplasm in keratinocytes treated with dimethyl sulfoxide (DMSO) control; (**A2**) Nuclear 4′,6-diamidino-2-phenylindole (DAPI) staining of A1; (**B1**) Nuclear translocation of AHR is observed in keratinocytes treated with 100 μg/mL *Rhodiola crenulata* extract (RCE); (**B2**) Nuclear DAPI staining of B1; (**C1**) Isotype-matched negative control showed no positive staining; (**C2**) Nuclear DAPI staining of C1. Scale bar: 25 μm; (**D**) RCE increases the expression of *CYP1A1*. White bar expresses DMSO control. Data are expressed as mean ± SEM (*n* = 3). *: *p* < 0.05.

We first examined the cytotoxic effect of RCE on human keratinocytes. As shown in Supplementary Figure S1, RCE did not affect their survival at concentrations less than 150 μg/mL. We then evaluated the agonistic activity of RCE on AHR. In control keratinocytes, AHR was present mainly in the cytoplasm (Figure 1(A1,A2)). RCE (100 μg/mL) appeared to induce the cytoplasmic-to-nuclear translocation of AHR (Figure 1(B1,B2)). Isotype-matched negative control showed no positive staining (Figure 1(C1,C2)). In parallel with this, 10 to 100 μg/mL RCE upregulated *CYP1A1* expression (Figure 1D).

### 2.2. RCE Upregulates FLG, LOR, and IVL Expression in an AHR-Dependent Fashion

**Figure 2:**
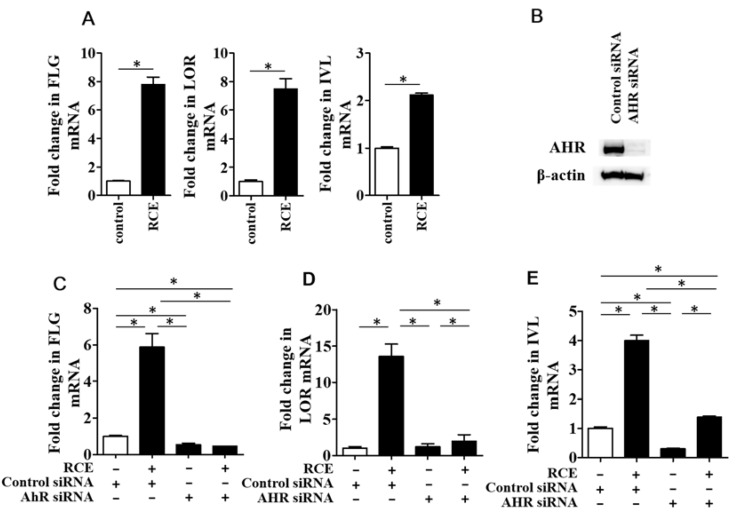
(**A**) RCE significantly increases the expression of filaggrin (*FLG*), loricrin (*LOR*), and involucrin (*IVL*) compared to DMSO control; (**B**) Protein level of AHR is downregulated in the keratinocytes transfected with AHR siRNA; (**C**) RCE-mediated *FLG* upregulation is canceled in AHR-knockdown keratinocytes transfected with AHR siRNA; (**D**) RCE-mediated *LOR* upregulation is canceled in AHR-knockdown keratinocytes; (**E**) RCE-mediated *IVL* upregulation is canceled in AHR-knockdown keratinocytes. White bar expresses DMSO control. Data are expressed as mean ± SEM (*n* = 3). *: *p* < 0.05. RCE = 100 μg/mL.

**Figure 3:**
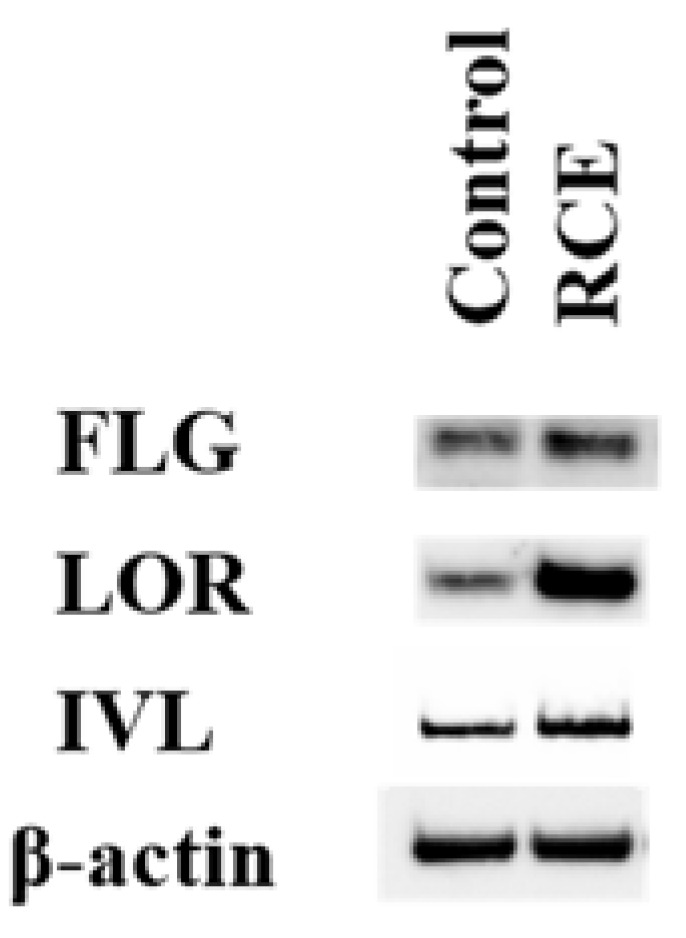
RCE upregulates the protein expression of FLG, LOR, and INV compared to DMSO control. RCE = 100 μg/mL.

The agonistic activation of AHR has been reported to upregulate *FLG, LOR*, and *IVL* expression [6,7,20]. In accordance with these previous studies, RCE significantly upregulated the expression of *FLG*, *LOR*, and *IVL* in this study (Figure 2A). In order to know the AHR dependency, we used AHR siRNA. As shown in Figure 2B, protein level of AHR was successfully downregulated in the keratinocytes transfected with AHR siRNA. The upregulating activity of RCE on *FLG*, *LOR*, and *IVL* expression was canceled in the AHR-knockdown keratinocytes transfected with AHR siRNA (Figure 2C–E), confirming the dependence of barrier protein expression on AHR. We next confirmed the protein levels of these barrier proteins by Western blot analysis. As shown in Figure 3, RCE increased the protein levels of FLG, LOR, and IVL compared to dimethyl sulfoxide (DMSO) control.

### 2.3. RCE Upregulates OVOL1 Expression in an AHR-Dependent Manner

**Figure 4:**
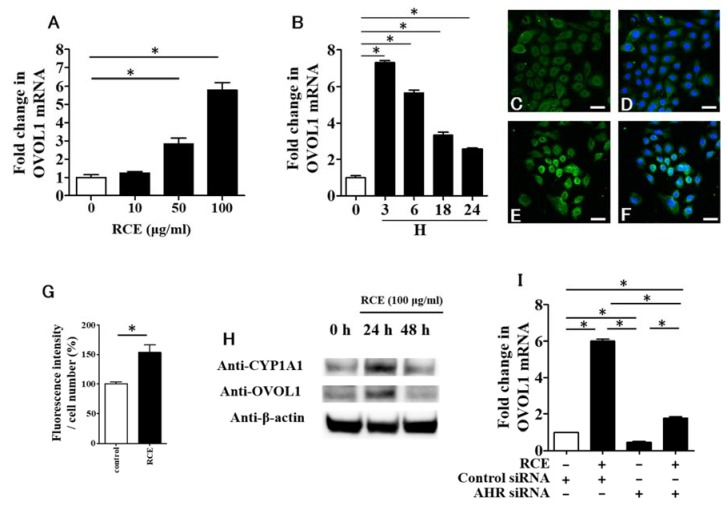
(**A**) RCE upregulates OVO-like 1 (*OVOL1*) expression; (**B**) RCE (100 μg/mL) upregulates *OVOL1* expression as early as 3 h after the treatment; (**C**,**D**) OVOL1 is mainly localized in the cytoplasm of keratinocytes treated with DMSO control; (**D**) Nuclei are stained with DAPI; (**E**,**F**) Nuclear translocation of OVOL1 is observed in keratinocytes treated with RCE (100 μg/mL); (**F**) Nuclei are stained with DAPI; Scale bar: 25 μm; (**G**) Fluorescence intensity of OVOL1 is enhanced in keratinocytes treated with RCE (100 μg/mL); (**H**) Upregulation of OVOL1 as well as CYP1A1 protein by RCE (100 μg/mL) is shown by Western blot analysis; (**I**) RCE-mediated *OVOL1* expression is canceled in AHR-knockdown keratinocytes. White bar expresses DMSO control. Data are expressed as mean ± SEM (*n* = 3). *: *p* < 0.05.

Our previous studies identified that OVOL1 is required in *FLG* and *LOR* upregulation [11,12]. In accordance with this, RCE also upregulated OVOL1 expression in this study (Figure 4A). The upregulation of OVOL1 mRNA was rapid and robust at a stage as early as 3 h, and gradually declined until 24 h after the incubation with RCE (Figure 4B). Immunofluorescence study revealed that OVOL1 was mainly located in the cytoplasm of monolayer keratinocytes (Figure 4C,D). In the presence of RCE, nuclear translocation of OVOL1 was evident with increased intensity of OVOL1 fluorescence (Figure 4E–G). Western blot analysis also confirmed the upregulation of protein levels of OVOL1 as well as CYP1A1 at 24 h after RCE treatment (Figure 4H). The OVOL1 upregulation by RCE was canceled in AHR-knockdown keratinocytes, confirming again its dependence on AHR (Figure 4I).

### 2.4. RCE-Mediated IVL Upregulation Is OVOL1-Independent

**Figure 5:**
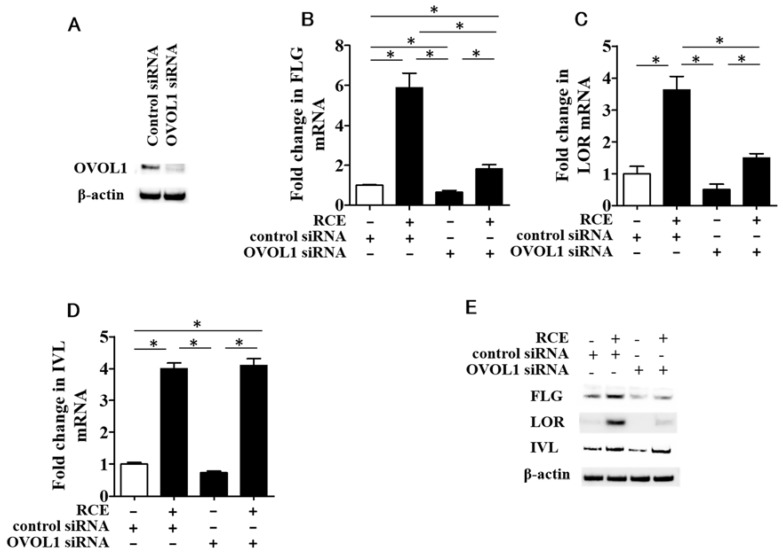
(**A**) Protein level of OVOL1 is downregulated in keratinocytes transfected with OVOL1 siRNA; (**B**) RCE-induced *FLG* upregulation is canceled in OVOL1-knockdown keratinocytes; (**C**) RCE-induced *LOR* upregulation is canceled in OVOL1-knockdown keratinocytes; (**D**) RCE-induced *IVL* upregulation is not canceled in OVOL1-knockdown keratinocytes; (**E**) The RCE-induced *FLG* and *LOR*, but not *IVL*, protein upregulation is canceled in keratinocytes transfected with OVOL1 siRNA. White bar expresses DMSO control. Data are expressed as mean ± SEM (*n* = 3). *: *p* < 0.05. RCE = 100 μg/mL.

We next examined whether OVOL1 regulates the upregulation of FLG, LOR, and IVL. Protein level of OVOL1 is downregulated in the keratinocytes transfected with OVOL1 siRNA (Figure 5A). As demonstrated for other AHR agonists [11,12], the RCE-mediated *FLG* (Figure 5B) and *LOR* (Figure 5C) upregulation was canceled in OVOL1-knockdown keratinocytes transfected with OVOL1 siRNA. However, unexpectedly, the RCE-mediated *IVL* upregulation (Figure 5D) was not altered in the OVOL1-knockdown keratinocytes. In parallel with gene expression, RCE upregulated the protein expression of FLG, LOR, and IVL (Figure 5E). The RCE-mediated FLG and LOR, but not IVL, protein upregulation was canceled in the OVOL1-knockdown keratinocytes (Figure 5E).

## 3. Discussion

### 3.1. Barrier Function and AHR-OVOL1 Axis

AHR plays a pivotal role in constituting the barrier between the environment and the body [3,21,22,23,24,25]. The intake of AHR ligands is essential for the correct development of intestinal innate immunity in infants [21]. The intestinal microbiome is also actively involved in producing various AHR ligands [21,22]. Impairment of proper ligation of AHR leads to the development of colitis [21,22]. Similar to the case in the intestine, AHR is also an indispensable sensor and transcription factor for orchestrating the barrier function of the skin [4,6,8]. Various bioproducts from the commensal microbiome and tryptophan photoproducts are AHR ligands [26,27]. Many phytoproducts used in folk medicine also contain various ligands for AHR, which upregulate FLG expression and improve the skin barrier function [28,29]. Recently, Tsuji et al. revealed that AHR-induced FLG and LOR upregulation is mediated by OVOL1 [11,12]. OVOL1 is a critical transcription factor for keratinocyte differentiation [13,14,15,16]. Notably, both *FLG* and *OVOL1* were included among 31 genes identified as being significantly linked to susceptibility to atopic dermatitis by genome-wide association studies [30,31,32,33,34,35,36].

### 3.2. Upregulation of FLG, LOR, and IVL by AHR Activation

The expression of *FLG*, *LOR*, and *IVL* is downregulated in the lesional skin of atopic dermatitis [9,37,38,39] and is restored by appropriate treatments [9,38,39]. In addition, some phytochemicals were reported to upregulate the FLG expression that is associated with accelerated barrier recovery in a tape-stripped, barrier-disrupted animal model [28,29]. As *Rhodiola* species exhibit skin-protecting effects against ultraviolet and γ-radiation, we speculated that RCE may possess agonistic activity for AHR.

### 3.3. Differential Regulation of FLG, LOR, and INV by AHR-OVOL1 Signaling

**Figure 6:**
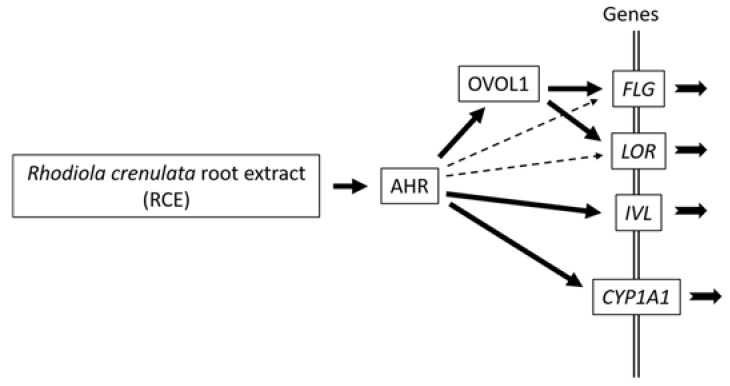
Differential regulation of skin barrier proteins by *Rhodiola crenulata* root extract. RCE is a potent activator of AHR. Ligation of AHR by RCE upregulates gene and protein expression of *CYP1A1* as well as skin barrier proteins such as FLG, LOR, and IVL. The upregulation of *CYP1A1* and IVL is directly regulated by AHR. However, the FLG and LOR upregulation is mainly regulated by AHR-OVOL1 signaling (solid arrows). However, there may exist another pathway in which AHR directly upregulates FLG and LOR expression (dotted arrows). Coordinated upregulation of skin barrier proteins by RCE may be beneficial for the management of barrier-disrupted inflammatory skin diseases such as atopic dermatitis.

In this study, RCE activated AHR, induced its cytoplasmic-to-nuclear translocation in keratinocytes, and upregulated the expression of CYP1A1, a specific AHR-responsive gene. Moreover, RCE upregulated gene and protein expression of FLG, LOR, and IVL expression. The FLG and LOR upregulation was mediated via the AHR-OVOL1 pathway. However, this was not the case for IVL expression. The RCE-mediated IVL upregulation was dependent on AHR, but was independent of OVOL1. These results indicate (1) that AHR ligation induces IVL, FLG, and LOR expression; (2) that the activation of AHR upregulates IVL expression (early-phase terminal differentiation protein) without help from OVOL1; and (3) that FLG and LOR expression (late-phase terminal differentiation proteins) is mediated by the AHR-OVOL1 pathway (Figure 6). Skin barrier disruption initiates and exacerbates inflammatory skin diseases such as atopic dermatitis [6,40]. Recent clinical trials have shown that the topical AHR agonist tapinarof successfully improves clinical symptoms of atopic dermatitis and psoriasis [41,42,43]. Topically applied, RCE is thus potentially beneficial for the treatment of barrier-disrupted skin conditions via its capacity to upregulate barrier proteins.

## 4. Materials and Methods

### 4.1. Reagents and Antibodies

*Rhodiola crenulata* extract (RCE) was provided by Procter and Gamble Innovation Godo Kaisha (Kobe, Japan) as 70% ethanol extract powder. Dimethyl sulfoxide (DMSO) was purchased from Nacalai Tesque (Kyoto, Japan). *Rhodiola crenulata* extract was dissolved in DMSO and stored at −30 °C until used in the experiments. 

Anti-AHR rabbit polyclonal antibody, anti-FLG mouse monoclonal, and normal mouse and rabbit IgG were purchased from Santa Cruz Biotechnology (Dallas, TX, USA). Anti-OVOL1 polyclonal rabbit antibody (LifeSpan Biosciences, Seattle, WA, USA) was used for immunofluorescence staining. Anti-OVOL1 monoclonal mouse antibody (Abcam, Cambridge, UK), anti-*CYP1A1* polyclonal mouse antibody (Abcam), anti-LOR polyclonal rabbit antibody (Abcam), anti-IVL monoclonal mouse antibody (Abcam), and anti-β-actin monoclonal mouse antibody (Cell Signaling Technology, Danvers, MA, USA) were used for western blotting.

### 4.2. Cell Culture

Normal human epidermal keratinocytes (NHEKs) obtained from Lonza (Walkersville, MD, USA) were grown in culture dishes at 37 °C in 5% CO_2_. They were cultured in serum-free keratinocyte growth medium (Lonza) supplemented with bovine pituitary extract, recombinant epidermal growth factor, insulin, hydrocortisone, transferrin, and epinephrine. Culture medium was replaced every 2 days. Near confluence (70–90%), cells were disaggregated with 0.25 mg/mL trypsin/0.01% ethylenediaminetetraacetic acid and subcultured. Second- to fourth-passage NHEKs were used in all experiments. NHEKs (1 × 10^5^) were seeded in 24-well culture plates, allowed to attach for 24 h, and then subsequently treated with or without RCE and DMSO.

### 4.3. Immunofluorescence and Confocal Laser Scanning Microscopic Analysis

NHEKs (2 × 10^4^) were cultured on slides (Lab-Tek, Rochester, NY, USA) with or without RCE for 5 h. The slides were then washed in phosphate-buffered saline (PBS), fixed with acetone for 10 min, and blocked using 10% bovine serum albumin (Roche Diagnostics, Basel, Switzerland) in PBS for 30 min. Samples were incubated with primary anti-AHR (1:50) or anti-OVOL1 (1:50) antibody in Western breeze blocker/diluent (Invitrogen, Carlsbad, CA, USA) overnight at 4 °C. The slides were then washed with PBS before incubation with anti-rabbit secondary antibody (Alexa Fluor 546 or 488; Molecular Probes, Eugene, OR, USA) for 1 h at room temperature. After nuclear staining with 4′,6-diamidino-2-phenylindole (DAPI), the slides were mounted with UltraCruz mounting medium (Santa Cruz Biotechnology). All samples were analyzed using a D-Eclipse confocal laser scanning microscope (Nikon, Tokyo, Japan).

### 4.4. Reverse Transcription-PCR and qRT-PCR Analyses

Total RNA was extracted using the RNeasy Mini kit (Qiagen, Hilden, Germany). Reverse transcription was performed using PrimeScript RT-reagent kit (Takara Bio, Otsu, Japan). qRT-PCR was performed on the CFX connect real-time system (Bio-Rad, Hercules, CA, USA) using SYBR Premix Ex Taq (Takara Bio). Amplification was started at 95 °C for 30 s as the first step, followed by 40 cycles of qRT-PCR at 95 °C for 5 s and 60 °C for 20 s. mRNA expression was measured in triplicate and was normalized to β-actin expression levels. The primer sequences from Takara Bio and SABiosciences (Frederick, MD, USA) are shown in Supplementary Table S1.

### 4.5. Transfection with siRNAs against AHR and OVOL1

Small interfering RNAs (siRNAs) against AHR (AHR siRNA, s1200) and OVOL1 (OVOL1 siRNA, s9939), as well as siRNA consisting of a scrambled sequence that would not lead to specific degradation of any cellular message (control siRNA), were purchased from Ambion (Austin, TX, USA). NHEKs cultured in 24-well plates were incubated with a mixture of HiPerFect Transfection reagent (Qiagen) containing 5 nM siRNA and 3 μL of HiPerFect reagent in 0.6 mL of culture medium. After a 48 h incubation period, siRNA-transfected NHEKs were treated with RCE or left untreated for 24 h. The transfection of siRNA had no effect on cell viability, as demonstrated by microscopic examination. Knockdown efficiency was 91.9 ± 2.1% by AHR siRNA and 71.5 ± 2.4% by OVOL1 siRNA.

### 4.6. Western Blot Analysis

NHEKs were incubated for 5 min in lysis buffer (Complete Lysis M; Roche Diagnostics). The lysate protein concentration was measured with a BCA protein assay kit (Thermo Scientific, Rockford, IL, USA). Equal amounts of protein (10 μg for LOR and IVL; 40 μg for others) were dissolved in NuPage LDS sample buffer (Invitrogen) and 10% NuPage sample reducing agent (Invitrogen). Lysate were boiled at 70 °C for 10 min and loaded and run on 4–12% Bis-Tris Gel (Invitrogen) at 200 V for 20 min. The proteins were transferred to PVDF membrane (Invitrogen) and blocked in Western breeze blocker/diluent (Invitrogen). Membranes were probed with anti-*CYP1A1*, anti-OVOL1, anti-FLG, anti-LOR, anti-IVL, or anti-β-actin antibodies overnight at 4 °C. Anti-mouse and anti-rabbit horseradish peroxidase-conjugated IgG antibodies (Cell Signaling Technology) were used as secondary antibodies. Visualization of protein bands was accomplished with SuperSignal West Pico Chemiluminescent Substrate (Thermo Scientific) using the ChemiDoc Touch Imaging System (Bio-Rad).

### 4.7. Statistical Analysis

Unpaired Student’s *t*-test (when two groups were analyzed) and one-way ANOVA (for three or more groups) were used to analyze the results, with a *p*-value less than 0.05 being considered to indicate a statistically significant difference.

## Supplementary Materials

Supplementary materials can be found at http://www.mdpi.com/1422-0067/19/6/1654/s1.

## Abbreviations

AHR	Aryl hydrocarbon receptor
*CYP1A1*	Cytochrome P450 1A1
FLG	Filaggrin
IVL	Involucrin
LOR	Loricrin
NHEK	Normal human epidermal keratinocyte
OVOL1	OVO-like 1
RCE	*Rhodiola crenulata* root extract
