# Supplementary material for: Upregulation of FLG, LOR, and IVL Expression by Rhodiola crenulata Root Extract via Aryl Hydrocarbon Receptor: Differential Involvement of OVOL1
Source: Int J Mol Sci. 2018 Jun 4;19(6):1654. doi: 10.3390/ijms19061654 (PMC6032276; doi:10.3390/ijms19061654)
Supplement: Supplementary file 1 [file ijms-19-01654-s001.pdf]

Supplementary Materials: Upregulation of FLG, LOR, and IVL Expression by *Rhodiola crenulata* Root Extract via Aryl Hydrocarbon Receptor: Differential Involvement of OVOL1

Akiko Hashimoto-Hachiya, Gaku Tsuji, Mika Murai, Xianghong Yan and Masutaka Furue

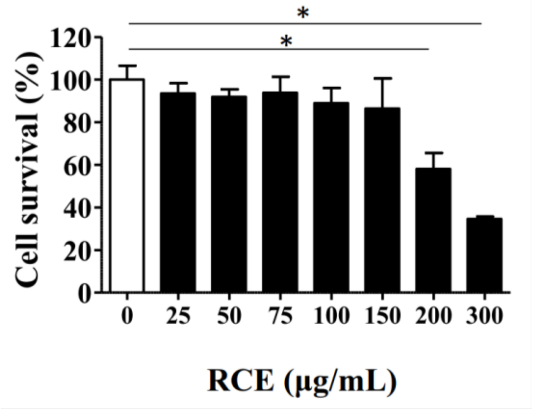

Figure S1. Cell viability analysis of normal human epidermal keratinocytes (NHEKs) is performed using a WST-1 assay (Takara Bio). NHEKs are treated with various concentrations of RCE (0, 25, 50, 75, 100, 150, 200, and 300 mg/mL) for 24 h. The formazan dye produced by viable cells is quantified by measuring the absorbance at $\lambda = 450$ nm. Data are shown in mean ± SEM. *: $p < 0.05$ ($n = 3$ for each group).

Table S1. Primers for qRT-PCR.

Gene	Forward Primer	Reverse Primer
OVOL1	5′-ACGATGCCCATCCACTACCTG-3′	5′-TTTCTGAGGTGCTGGTCATCATTC-3′
FLG	5′-CATGGCAGCTATGGTAGTGCAGA-3′	5′-ACCAAACGCACTTGCTTTACAGA-3′
LOR	5′-GAGTTGGAGGTGTTTTCCAGGG-3′	5′-GCAGAACTAGATGCAGCCGGA-3′
IVL	5′-TAACCACCCGCAGTGTCCAG-3′	5′-ACAGATGAGACGGGCCACCTA-3′
CYP1A1	PPH01271E (SABiosciences)	
β-actin	5′-ATTGCCGACAGGATGCAGA-3′	5′-GAGTACTTGCGCTCAGGAGGA-3′
